# Getting a tool gives wings even in schizophrenia: underestimation of tool-related effort in a motor imagery task

**DOI:** 10.1038/s41537-021-00175-y

**Published:** 2021-09-15

**Authors:** Amandine Décombe, Lionel Brunel, Vincent Murday, François Osiurak, Delphine Capdevielle, Stéphane Raffard

**Affiliations:** 1 University Department of Adult Psychiatry, Hôpital la Colombière, University Hospital of Montpellier, Montpellier, France; 2 Department of Psychology, Epsylon, Paul Valery University of Montpellier, Montpellier, France; 3 EMC Laboratory, Lyon Lumière University, Lyon, France; 4 Inserm, Unit 1061, Neuropsychiatry, Epidemiology and Clinical Research, University of Montpellier, Montpellier, France

**Keywords:** Schizophrenia, Human behaviour

## Abstract

Humans frequently use tools to reduce action-related efforts. Interestingly, several studies have demonstrated that individuals had tool-related biases in terms of perceived effort reduction during motor imagery tasks, despite the lack of evidence of real benefits. Reduced effort allocation has been repeatedly found in schizophrenia, but it remains unknown how schizophrenia patients perceive tool-related benefits regarding effort. Twenty-four schizophrenia patients and twenty-four nonclinical participants were instructed to move the same quantities of objects with their hands or with a tool in both real and imagined situations. Imagined and real movement durations were recorded. Similarly to nonclinical participants, patients overestimated tool-related benefits and underestimated tool-related effort in terms of time when they mentally simulated a task requiring the use of a tool. No association between movement durations and psychotic symptoms was found. Our results open new perspectives on the issue of effort in schizophrenia.

## Introduction

To achieve a goal, humans tend to adopt strategies to minimize the effort associated with a given action. One of the most efficient strategies is to design and use a tool. Therefore, in everyday life, humans frequently use tools to interact and modify their environment. Tools are defined as “objects amplifying the user’s sensorimotor capabilities, allowing the user to reduce the various costs (e.g., effort, time) required to accomplish a given task”^1–3^. Contrary to what have long been thought, tool use in not a unique capacity of humans, but can also be found in several animal species^1,2,4^. For instance, New Caledonian crows frequently make and use a variety of tools such as hooks or sticks to extract wood-boring beetle larvae in order to facilitate capturing prey^5,6^. Contrary to animals^4^, humans may be biased in the way they perceive tool-related efforts, particularly in overestimating tool-related benefits in terms of effort reduction^3^. Thus, although tool use is far from being unique to humans^5^, humans may be unique in their way of using tools or, more particularly, in how they perceive the efforts related to tool use. Hence, the issue of effort or energy expenditure might be crucial in our understanding of human tool use.

Past research indicates that the overestimation of the advantage provided by a tool is integrated in a number of ways within perceptual and decisional processes^3,7–11^. For instance, participants who were previously exposed to the benefits of using a tool in order to reach a target (i.e., using a stick for collecting tokens outside the participants’ arms’ reach) overestimated their reaching space^7^. Moreover, several studies have demonstrated that individuals had tool-related biases in terms of effort reduction during motor imagery^3,8,10–12^. For instance, Osiurak et al.^3^ examined the estimation of the benefits provided by a tool by measuring imagined and real movement durations in a sample of undergraduate students. The participants had to actually move, or to imagine moving (real vs. imagined situations), different quantities of objects with their hands (hand condition) or with a tool (tool condition) from one location to another. The tool was not available to the participants; they had to pick it up from a table. In some trials, tool use (i.e., tool actions) provided clear time-related benefits in comparison with the use of the hands (i.e., non-tool actions), whereas it was the opposite in other trials. Results revealed that participants showed a time-related bias in the imagined situation; the participants imagined spending less time using a tool than using their hands, compared to the actions they actually performed. This was particularly true for trials where tool use did not provide time benefits, as if participants anticipated that they would need less effort by using a tool, resulting in an effort bias toward the tool. To summarize, tool use or imagining using a tool have been repeatedly associated with an effort-related bias toward the tool, even in situations in which the use of a tool does not provide clear and real benefits.

At the same time, aberrant effort-cost decision-making has emerged in recent years as a growing area of research in the context of mental disorders, particularly across psychotic disorders. In the past decade, several studies have found that individuals with schizophrenia presented a deficit of effort-allocation^13^. Rather than a global reduction of effort expenditure, there is a tendency for individuals with schizophrenia to choose a lower effort to receive a small monetary reward over a higher effort associated to a greater reward^14–20^. Furthermore, notwithstanding a higher probability of greater reward, individuals with schizophrenia abnormally choose the lower effort for the lower reward^14,15,18–21^. This difficulty in choosing effort in response to increasingly rewarding cues has been positively associated with negative symptoms, notably amotivation^15,16,20–25^. However, not all studies have shown significant associations between deficit of effort allocation and negative symptoms^14,18,26^. As stated above, tool use is considered a central aspect of human evolution, leading to extended motor and sensory capabilities and, consequently, it might be of particular interest for our comprehension of abnormal effort processes in mental disorders. However, no study so far has investigated the perception of tool-related benefits in terms of effort in schizophrenia.

The main objective of this study was therefore to explore effort-related bias toward tools in individuals with schizophrenia, compared to non-clinical participants. To achieve this objective, we replicated the motor imagery experiment of Osiurak et al.^3^ Individuals with schizophrenia and non-clinical controls were asked to move or imagine moving toilet rolls from one point (table A) to another (table B). Toilet paper rolls (hereinafter ‘rolls’) were chosen because they are light, easy to grip and transporting them does not involve any risk when moving. Six quantities of rolls were to be moved ranging from 4 to 24. Participants had to use or imagine using their hands (i.e., moving 2 rolls at a time) and with a tool that allowed moving 4 rolls at a time. The tool was not within reach of the participants, it was placed on another table (table C). Thus, it was more advantageous to move the three smallest amounts of rolls with the hands and more advantageous to pick up the tool and use it to move the three largest amounts of rolls. We measured the durations of the real and imagined actions. We also calculated the speed gain related to motor imagery in subtracting the real situation from the imagined situation. This allowed us to investigate whether individuals overestimate the benefit associated with tool use (i.e., moving the rolls with the tool—distance AB) and underestimated the costs related to the search of the tool (i.e., picking up the tool at the beginning and putting it down at the end—distance AC). Given that effort in the context of tool use has not been researched in the framework of schizophrenia yet, we did not develop specific hypotheses and consider it as exploratory. This experiment was part of a larger protocol comprising of another study about effort perception in schizophrenia^27^. Moreover, given that reduced willingness to exert effort in exchange for reward has been repeatedly associated with negative symptoms in schizophrenia^15,16,21,22^, our second objective was to study the relationship between effort-related bias and negative symptoms, particularly amotivation, in the context of tool use. Since we did not manipulate the reward (i.e., participants did not receive any reward for their participation) in contrast to past studies, statistical analyses between effort and negative symptoms were also considered as exploratory.

## Results

### Group comparisons

Table 1 compares sociodemographic, clinical, body weight (in kg), and neuropsychological data for the two groups. No differences were found with regards to gender, education, age, planning, and premorbid IQ. A significant difference was found in working memory; scores were lower in the schizophrenia group (*M* = 17.58, SD = 3.09) compared to the healthy control group (*M* = 20.75, SD = 3.95), *t*(46) = 3.23, *p* = 0.002. Body Mass Index (BMI) was higher in the schizophrenia group (*M* = 26.62, SD = 4.39) than in the non-clinical group (*M* = 22.37, SD = 2.86), *t*(46) = −3.97, *p* < 0.001. Equivalent chlorpromazine was positively correlated with bodyweight in the schizophrenia group (*r*(22) = 0.459, *p* = 0.024).

**Table 1:** Sociodemographic and clinical variables.

	Schizophrenia group	Healthy control group	Group comparisons	*P*-value
Total *N*	24	24		
Gender/ Male *N*(%)	17 (70.83)	17 (70.83)	*χ*² (1, *N*) = 0.00	*p* = 1.00
Age M(SD)	31.28 (7.42)	27.75 (4.94)	*t*(46) = −1.99	*p* = 0.052
Education M(SD)	12.88 (2.25)	13.54 (2.02)	*t*(46) = 1.08	*p* = 0.29
Symptoms				
PANSS Negative M(SD)	13.92 (5.66)			
PANSS Amotivation M(SD)	5.92 (3.02)			
Planning time M(SD)	431.54 (455.83)	351.92 (200.74)	*U* = 276.00, *z* = 0.24	*p* = 0.813
Planning errors M(SD)	3.38(1.193	2.46 (1.77)	*t*(46) = −1.72	*p* = 0.09
Working memory—raw score M(SD)	17.58 (3.09)	20.75 (3.95)	*t*(46) = 3.09	*p* = 0.003
fNART premorbid IQ M(SD)	106.52 (7.53)	108.11 (8.84)	*t*(46) = 0.70	*p* = 0.49
Weight (in kg) M(SD)	80.69	67.46 (12.49)	*t*(46) = −3.18	*p* = 0.003
Body Mass Index (BMI) M(SD)	26.62 (4.39)	22.37 (2.86)	*t*(46) = −3.97	*p* < 0.001
Antipsychotic dosage (CPZ Eq) M(SD)	547.92 (374.14)			

Mean, standard deviation, median, and range are presented for gender, age, education, clinical, neuropsychological, and body weight variables. Legend: *M* Mean, *SD* Standard Deviation, *Med* Median, *Rg* Range of value, Positive and Negative Symptoms Scale (PANSS), planning (revised shopping test, total time, errors), Working memory (Letter Digital Sequencing), Premorbid IQ (fNART, French version of National Adult Reading Test); Chlorpromazine equivalent dose (CPZ Eq). Group statistics were calculated with appropriate parametric vs. non-parametric tests.

### Preliminary analysis

Because the order (i.e., imagined first vs. real first) might influence movement durations^28,29^, we added the Order factor in our analyses beforehand. Order had no significant main or interaction effects on movement durations or imagery-related speed gain (all *ps* > 0.14), and thus was not considered in the main analysis.

Score in working memory was higher for individuals with schizophrenia, compared with nonclinical participants. Working memory as covariate had no significant main or interaction effects on movement durations or imagery-related speed gain (all *ps* > 0.13). Consequently, we did not integrate this covariate in subsequent analyses.

BMI may influence visual perception^30^ and is higher in the schizophrenia group compared to the healthy control group. BMI as covariate had no significant main or interaction effects on imagery movement durations or imagery-related speed gain (all *ps* > 0.32). Consequently, we did not integrate this covariate in subsequent analyses.

### Is using a tool less costly in terms of time? Movement durations in imagined vs. real situation

Analysis revealed a main effect of Group (*F*(1,46) = 4.43, *p* < 0.05, *η*²_*p*_ = 0.09). Individuals with schizophrenia were slower than non-clinical participants, in both conditions and in both situations.

A strong main effect of Rolls was found (*F*(1.41,64.82) = 789.02, *p* < 0.001, *η*²_*p*_ = 0.95). Post hoc multiple comparisons with Bonferroni corrections indicated that durations increased with the quantity of rolls to be moved (all *ps* < 0.001).

An interaction effect between Group and Rolls was also found (*F*(1.41,64.82) = 3.50, *p* < 0.01, *η*²_*p*_ = 0.07). After Bonferroni’s corrections, the post hoc tests revealed no significant difference between the two groups for each quantity of rolls (all *ps* > 0.12).

No main effect of Situation (*F*(1,46) = 0.37, *p* = 0.54, *η*²_*p*_ = 0.008) or Condition (*F*(1,46) = 0.53, *p* = 0.47, *η*²_*p*_ = 0.01) were found. Movement durations were not significantly different between imagined vs. executed situations, and between hands use vs. tool use.

However, we found a Situation x Condition effect (*F*(1,46) = 19.36, *p* < 0.001, *η*²_*p*_ = 0.30). Post hoc multiple comparisons with Bonferroni corrections indicated that participants imagined taking significantly less time to move the rolls with the tool compared to the executed situation of tool use (*p* < 0.01) and compared to imagining moving the rolls with their hands (*p* < 0.01).

A Condition x Rolls (*F*(1.96,90.35) = 109.89, *p* < 0.001, *η*²_*p*_ = 0.70) interaction effect was significant. Post hoc multiple comparisons with Bonferroni corrections indicated that participants executed or imagined taking less time with the hands to move 4 and 8 rolls (all *ps* < 0.001) compared to the tool, and less time with the tool to move 20 and 24 rolls compared with the hands (all *ps* < 0.001).

In addition, a Condition x Rolls x Group (*F*(1.96,90.35) = 3.16, *p* < 0.05, *η*²_*p*_ = 0.06) interaction effect was found. After Bonferroni’s corrections, the post hoc tests revealed no significant difference between the two groups for each quantity of rolls and each condition (all *ps* < 0.33).

**Fig. 1:**
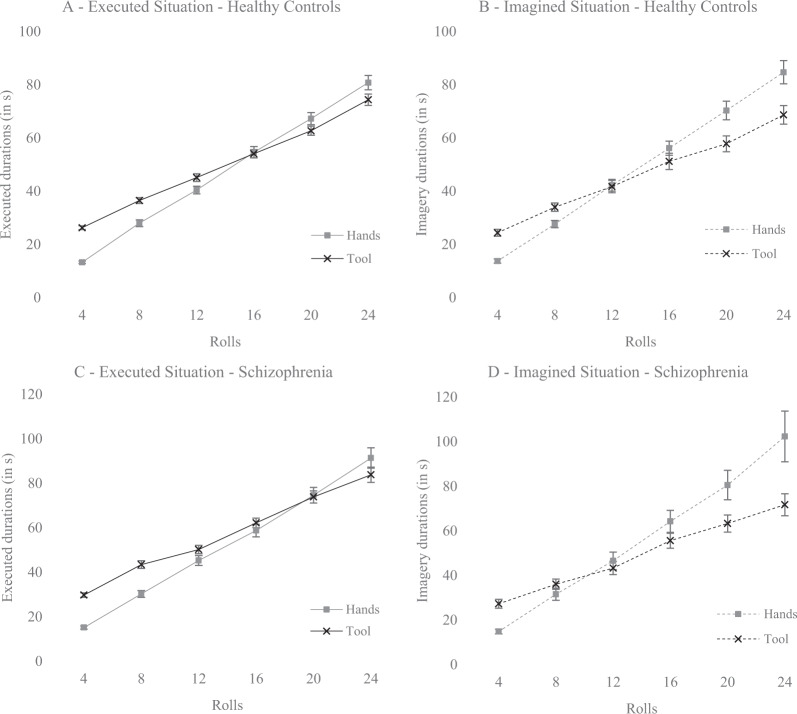
Movement durations as a function of rolls, conditions, situations, and groups. **A** Durations in real situation as a function of rolls and conditions in healthy control group. **B** Durations in imagined situation as a function of rolls and conditions in healthy control group. **C** Durations in real situation as a function of rolls and conditions in schizophrenia group. **D** Durations in imagined situation as a function of rolls and conditions in schizophrenia group (hands are represented by gray squares, tool by black crosses, and error bars correspond to standard errors).

Finally, the Situation x Condition x Rolls (*F*(1.83,84.29) = 5.46, *p* < 0.01, *η*²_*p*_ = 0.11) interaction effect was significant (see Fig. 1 for each group). Post hoc multiple comparisons with Bonferroni corrections indicated that, in the real situation, participants moved 4 and 8 rolls faster with the hands compared to the tool (all *ps* < 0.001) and moved 24 rolls faster with the tool compared to the hand (*p* < 0.01) in both groups. For 12, 16, and 20 rolls, no difference was found between the two conditions (hands vs. tool) in the real situation (all *ps* > 0.52). Concerning the imagined situation, multiple comparisons with Bonferroni corrections revealed that the individuals imagined moving 4 rolls faster with their hands compared to the tool (*p* < 0.001) and imagined moving 16, 20, and 24 rolls faster with using the tool compared to their hands (all *ps* < 0.01) in both groups. No significant difference was found between the two conditions (hands vs. tool) for 8 and 12 rolls in the imagined situation. The Situation x Condition x Rolls x Group interaction effect was not significant (*F*(1.83,84.29) = 1.19, *p* = 0.31, *η*²_*p*_ = 0.03), suggesting that both groups had a similar performance. No Situation x Group (*F*(1,46) = 0.04, *p* = 0.85, *η*²_*p*_ < 0.001), Condition x Group (*F*(1,46) = 0.28, *p* = 0.60, *η*²_*p*_ = 0.006), Situation x Rolls (*F*(1.62,74.70) = 0.18, *p* = 0.79, *η*²_*p*_ = 0.004), Situation x Condition x Group (*F*(1,46) = 2.82, *p* = 0.10, *η*²_*p*_ < 0.06), or Situation x Rolls x Group (*F*(1.62,74.70) = 0.10, *p* = 0.87, *η*²_*p*_ = 0.002) interaction effects were found.

Similar to the results of Osiurak et al.^3^, the durations in the real situation did not match the predicted distances (see Table 3). In addition, results showed an advantage in terms of time for tool use in the imagined situation compared to the real situation. For these reasons, and to see which distance (AB, AC, or both) was underestimated in terms of time, we calculated a measure of speed gain related to the imagery.

### Overestimation of benefits, underestimation of effort, or both: imagery-related speed gain

**Fig. 2:**
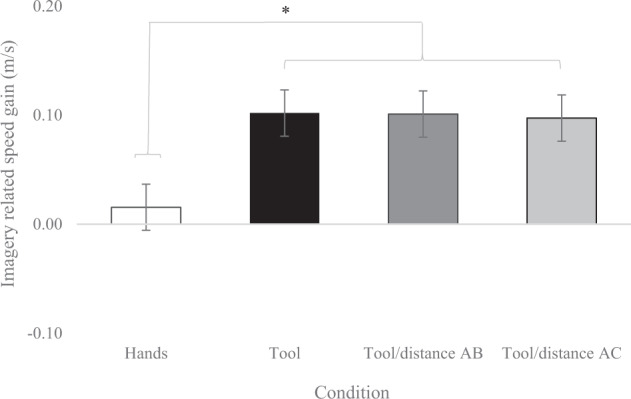
Imagery-related speed gain as a function conditions in both groups. Stars indicate significant post-hoc differences—Student’s *t* tests—with a level of significance of *p* < 0.05, corrected using the Bonferroni procedure. A positive score indicated that the participants took less time to imagine the actions compared to the execution of the actions (hands are represented in white, tool in black, tool/distance AB in dark gray, tool/distance AC in light gray, and error bars correspond to standard errors.

The main effect of Group (*F*(1,46) = 0.70, *p* = 0.41), the main effect of Rolls (*F*(2.60,119.48) = 0.58, *p* = 0.71), Group x Rolls (*F*(2.60,119.48) = 0.61, *p* = 0.59, *η*²_*p*_ = 0.01), Group x Condition (*F*(1.82,83.84) = 0.31, *p* = 0.71, *η*²_*p*_ = 0.007), Condition x Rolls (*F*(5.42,249.44) = 0.58, *p* = 0.73, *η*²_*p*_ = 0.01), and Group x Condition x Rolls (*F*(5.42,249.44) = 1.40, *p* = 0.22, *η*²_*p*_ = 0.03) interaction effects were not significant. ANOVA revealed only a strong main effect of Condition (*F*(1.82,83.84) = 12.25, *p* < 0.001, *η*²_*p*_ = 0.21). Post hoc multiple comparisons with Bonferroni corrections indicated that the conditions of tool, AB/tool, and AC/tool were significantly higher than hands (all *ps* < 0.007). The tool conditions (tool, AB/tool, AC/tool) did not differ significantly from each other (all *ps* > 0.90, see Fig. 2). Both groups imagined moving faster to get the tool (i.e., AC distance) and to move the rolls with the tool (i.e., AB distance) faster than they really did. Regarding the use of the hands, the speed gain score related to imagery was close to zero; all individuals imagined moving the rolls at the speed they actually did in the real situation.

### Correlations with amotivation in schizophrenia

Spearman correlations are presented in Table 2. With regards to imagery-related speed gain, no significant correlation was found with amotivation (all *ps* > 0.31).

**Table 2:** Spearman correlations.

Imagery-related speed gain		PANSS negative	PANSS amotivation
Tool	*r*(22)	−0.013	0.048
	*p*	0.95	0.82
Tool AC—Cost-related tool	*r*(22)	−0.061	−0.039
	*p*	0.78	0.86
Tool AB—Benefit-related tool	*r*(22)	0.011	0.086
	*p*	0.96	0.69
Hands	*r*(22)	0.100	0.127
	*p*	0.64	0.55

## Discussion

It has been shown that people overestimate the benefits provided by tool use in terms of cost-benefits, which leads individuals to use a tool even though it objectively provides less time-based benefits than using one’s own hands. The aim of this study was to investigate whether individuals with schizophrenia overestimated tool-related benefits in a motor imagery task, as it has been previously found in healthy controls. To that end, we replicated the motor imagery experiment of Osiurak et al.^3^ in a group of schizophrenia patients and compared them to heathy participants. Movement durations were collected in function of situation (real vs. imagined), condition (hands vs. tool), and quantity of rolls to be moved (6 different quantities). Moreover, we computed an imagery-related speed gain according to each distance. Thus, using a tool can be considered under two aspects: cost (distance AC—searching for the tool) and benefit (distance AB—using the tool to move the rolls).

First, we found that individuals with schizophrenia were overall slower than non-clinical individuals in both situations (imagined and real), independently of conditions (hand vs. tool) or quantities of rolls to be moved. Our results are in line with previous findings demonstrating motor abnormalities and, particularly, a speed reduction in both gait^31,32^ and motor imagery of gait^33^ in schizophrenia. Psychomotor slowing is also found in fine motor tasks such as writing^34^ and finger tapping^35^. Motor abnormalities including psychomotor slowing are central features of schizophrenia and may even be one of its biomarkers^36^. Thus, our results extend psychomotor slowing in the case of tool use in schizophrenia.

Second, we found that in imagined situations, both groups imagined taking less time for tool use than they really did. Indeed, both groups imagined moving the three largest quantities of rolls faster with the tool than with their hands whereas, in the real situation, both groups were faster with the tool only when moving the largest quantity of rolls. This result indicated that, despite the psychomotor slowing, individuals with schizophrenia perceived tool use-related actions as less costly in terms of time, as the healthy control participants did. Even with the presence of psychomotor slowing, our results indicated that individuals with schizophrenia perceived a reduction in time associated with tool use. We consequently generalized the findings of Osiurak et al.^3^ to a group of individuals with schizophrenia. In a nonverbal social perception context, Walther et al.^37^ found impaired tool-use performance (i.e., scoop and hammer) in schizophrenia. Performance was assessed through grip formation, execution, direction, and space of tool use. In our study, we did not measure the accuracy of tool use but rather the duration to perform the tool-related action. Thus, although individuals with schizophrenia were less accurate in tool use, they were able to perceive the time reduction associated with tool use. The effort reduction component associated with tool use appears to be preserved in schizophrenia. In addition, the consistency of our results in non-clinical individuals with those of Osiurak et al.^3^ highlights the robustness of this paradigm. A possible interpretation could be that tool use allows individuals with schizophrenia, as well as non-clinical individuals, to imagine gaining more time. Time gain is a sub-criterion of perceived usefulness, explaining the intention of using a tool in the technology acceptance model^38^. This time gain is an argument in favor of frequent tool use in healthy controls and also in individuals with schizophrenia.

Third, regarding tool-related costs, imagery-related speed gain results showed that patients imagined taking less time than they actually did to pick up the tool at the beginning and to drop it off at the end of the trial (i.e., AC distance). This finding showed that individuals with schizophrenia tended to underestimate tool-related costs, as healthy controls did. In Osiurak et al.^3^, individuals also tended to underestimate the cost. Our results also generalize those of Osiurak et al.^3^ in the schizophrenia population. This adds a new understanding to the processes related to effort in schizophrenia. Previous studies highlighted an effort allocation deficit in schizophrenia^13^. Specifically, in decision-making tasks, individuals with schizophrenia were less willing to exert a high effort for a greater reward than non-clinical individuals. In this study, we tested another effort-related process through motor imagery, i.e., anticipation of effort. This anticipatory process is crucial for decision-making. Prior to making a conscious decision, specific brain areas are activated to provide information predicting the outcome of a motor decision^39^. We showed for the first time that individuals with schizophrenia were able to anticipate a reduction in tool-associated effort in a similar way to healthy controls. The mechanism of effort anticipation, preceding decision-making, seems to be efficient in schizophrenia.

Regarding tool-related benefit, individuals with schizophrenia imagined that they were faster than they really were to move the rolls with the tool (i.e., AB distances). Patients tended to overestimate the benefit provided by tool-use, similarly to healthy controls. Individuals with schizophrenia could integrate the benefits of the tool into their mental representation, as could non-clinical individuals. Nevertheless, this result was not found in Osiurak et al.^3^, although they hypothesized an overestimation of the tool-related benefit in terms of time. Our result provides new insights into the understanding of cost-benefit computations in schizophrenia. Previous studies, which showed a deficit of effort-based decision-making, always manipulated effort with monetary reward^13^. Monetary reward was a benefit and a consequence to effort allocation. In our study, we did not manipulate monetary reward, but the benefit associated to tool use. It is a benefit which is directly related to actually performing the action. Consequently, in the absence of reward, our results indicate that individuals with schizophrenia were able to perceive benefit, if it is directly related to the action, similarly to healthy controls.

In addition, regarding our second objective about the relationship between anticipation of effort and negative symptoms, such as amotivation, we found no significant association in the schizophrenia group. This lack of significant results may be due to the low severity of negative symptoms in our sample. Previous studies which did not find a significant association also share this limitation of minimally symptomatic participants^14,18,26^. Alternatively, it is possible that tool use, in its anticipatory component (i.e., motor imagery), is not related to negative symptoms. Future studies based on samples with higher severity of negative symptoms are needed to address this issue. Overall, these findings confirm the results of Osiurak et al.^3^, indicating that healthy people perceive tool actions as less costly in terms of movement duration than they actually are, and can be generalized to individuals with schizophrenia. In addition, our finding suggests that, through motor imagery, individuals with schizophrenia can anticipate the reduction in effort that tool use brings, in a similar way to non-clinical individuals. In view of the motivational difficulties in schizophrenia, and in particular the allocation of effort for reward, our results provide new clinical perspectives. Specifically, if individuals with schizophrenia anticipate the reduction in effort provided by the tool, they may then use it frequently in order to mobilize less effort. However, in our study, the two groups did not have the choice of using the tool or their hands; they passed both conditions. Therefore, we cannot conclude on tool preference in schizophrenia, although they do perceive a benefit (i.e., reduced effort). For future studies, it would be interesting to test tool-based decision-making in schizophrenia, such as Experiment 2 by Osiurak et al.^3^. Nevertheless, our study allows us to conclude that our results satisfy a technology acceptance sub-criterion, i.e., the perceived time gain from tool use.

Several limitations should be noted. As mentioned above, our sample sizes were modest in both groups, which may contribute in part to the lack of significant associations between anticipated effort in terms of times and amotivation. It would have been interesting to compare other clinical measurements, such as the Global Assessment of Functioning. Indeed, we did not find any significant association with negative symptoms and our measurements related to effort, which is likely due to a relatively symptomless sample, or to the fact that the choice of measures for assessing negative symptoms was not the gold standard (i.e., PANSS). However, the reduction in effort reflected by the durations of tool-related actions could be related to functioning, as previously shown in effort allocation in schizophrenia^22,25^. Finally, and contrary to previous study^3^, we did not explore decision-making task related to tool use. Therefore, we cannot know whether individuals with schizophrenia explicitly prefer to choose a tool even though they implicitly perceive (i.e., motor imagery bias) the benefits of the tool use.

Tools in everyday life allow us to minimize effort. As such, and as postulated by some authors, “The more intelligent people are, the more they use tools”^40^. To date, most studies of effort in schizophrenia have focused on experimental effort decision-making paradigms. We believe that the study of tool use and its links with effort could open new perspectives, both theoretical and clinical, concerning the issue of effort investment in schizophrenia.

## Methods

### Participants

Twenty-five individuals with a diagnosis of either schizophrenia (*n* = 22) or schizoaffective disorder (*n* = 3) according to DSM 5 criteria and 25 healthy controls were recruited in the University Department of Adult Psychiatry of the Montpellier University Hospital, France. Participants were excluded if they presented: (1) neurological disorders; (2) cranial trauma antecedents; (3) substance use disorders (except for mild and moderate cannabis and tobacco use disorders). To ensure the absence of psychotic disorders in healthy control participants, we used the 7th version of the DSM 5 Mini-International Neuropsychiatric Interview. Two participants (1 healthy control and 1 individual with schizophrenia) were excluded because they did not finish the experiment. All participants were French and had the minimal reading level required by the French National Adult Reading test (f-NART^41^). Participants were matched for age, gender, and level of education (see Table 1). For patients, treatment doses were calculated as chlorpromazine equivalent according to the minimum effective dose method^42^. Prior to the experiment, all participants provided written informed consent, approved by the local Ethics Committee (CPP Sud Méditérannée III, Montpellier, France, 2020-A02404-35) and conforming to the Declaration of Helsinki.

### Clinical symptoms and neuropsychological measures

Psychotic and particularly negative symptoms were assessed with the Positive and Negative Syndrome Scale (PANSS^43^) for patients. We calculated the subdimension of negative symptoms, according to the PANSS five-factor model^44^. In addition, we also used the PANSS Amotivation factor^45,46^, which highly correlated with Brief Negative Symptoms Scale (BNSS^47^) and effort-based decision-making^48^. The following cognitive tests were administered to all participants: Letter Digit Sequencing span of WAIS-IV, revised shopping test^49^, and the fNART. The Letter Digit Sequencing span was used to measure mental charge in working memory and the participant’s ability to remember instructions. The revised shopping test measures planning. In this test, we measured the time the participants took to complete the test, and noted the ‘errors’, i.e., the total of the errors of the following types: useless detours, logical errors, and non-respect of the times. The fNART estimates verbal and premorbid IQ. We collected physical data (i.e., Body Mass Index, BMI) because it was higher in schizophrenia^50,51^ and it influences visual perception^30^.

### Experimental task

#### Apparatus

**Fig. 3:**
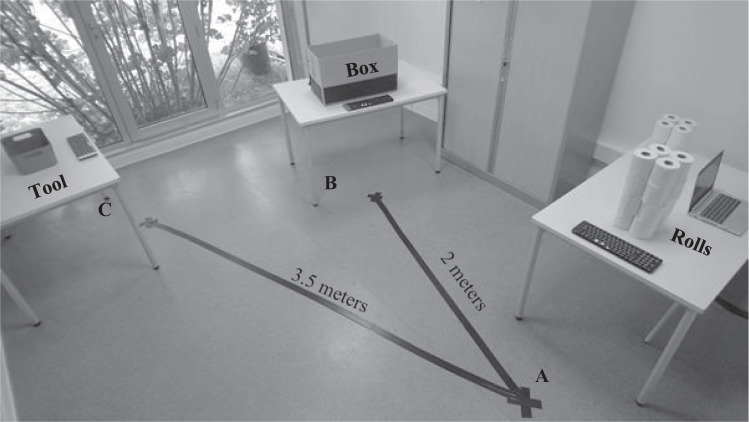
Experimental set up. The rolls were placed on table A, the box on table B, and the tool (i.e., the bowl) on table C. A Bluetooth keyboard was placed on each table. The distances were drawn on the floor, the distance AB was 2 m and the distance AC was 3.5 m.

Participants were instructed to move (i.e., real situation) or imagine moving (i.e., imagined situation) various quantities of rolls (4, 8, 12, 16, 20, 24), using their hands or a tool (i.e., a bowl). The apparatus consisted of three tables of the same size (length: 120 cm; width: 80 cm; depth: 80 cm), 24 rolls of toilet paper, a box (length: 92 cm; width: 64 cm; depth: 64 cm), a bowl (i.e., tool, length: 34 cm; width: 12 cm; depth: 22 cm), a computer, and three Bluetooth keyboards, with one on each table. A schematic representation is shown in Fig. 3.

Toilet paper rolls, a Bluetooth keyboard, and a computer were placed on Table A. On table B, there was a box to put the rolls in and a Bluetooth keyboard. On table C, we placed a bowl large enough to hold 4 rolls and light enough (204 g) to be easily handled and moved by the participants. To control the distances between the tables, lines were drawn on the floor (see Fig. 3). The AB and AC distances were 2 and 3.5 m, respectively. These two distances were chosen so that the hand condition required less time than the tool condition for the three smallest quantities of rolls (i.e., 4, 8, and 12 rolls), and vice versa for the three largest quantities of rolls (i.e., 16, 20, and 24 rolls) (see Table 3).

**Table 3:** Distance in function of the quantity of rolls and the condition.

Quantity of rolls	Hand condition			Tool condition		
	AB distance	AC distance	Total distance	AB distance	AC distance	Total distance
	2 m	3.5 m		2 m	3.5 m	
4	8	0	8	4	14	18
8	16	0	16	8	14	22
12	24	0	24	12	14	26
16	32	0	32	16	14	30
20	40	0	40	20	14	34
24	48	0	48	24	14	38

A computer equipped with the OpenSesame 3.0.7 software^52^ was placed on table A so as not to disturb the movements of the participants. The OpenSesame software was used to record participants’ movement durations (via Bluetooth keyboards) and to follow the movements of the participants.

### Experimental task

#### Real situation

Participants were asked to move the rolls under two conditions: either with their hands or with a tool. In the hands condition, the participants had to move the rolls from table A to the box on table B (see Fig. 3). Rolls could be moved two at a time. At the end of the trial, the participants had to return to table A. In the tool condition, at the beginning, the participants had to go to table C to take the tool, a bowl, and then had to return to table A. The rolls are moved four at a time using the bowl. Once the 4 rolls were in the bowl, the participants had to go to table B and throw the rolls into the box. When all the rolls were moved, the participants had to go to table C to put the bowl on the table, and return to table A. For all conditions, the lines marked on the floor had to be respected. Finally, participants were asked to press the spacebar on the keyboard each time they arrived at a table and end the task at table A. Real action duration was therefore recorded for each distance (AB, BA, AC, and CA).

#### Imagined situation

The imagined situation was the same as the real situation with regards to the hands and tools conditions, except that the participants had to imagine each action. They had to stay close to table A and to press the space bar on the keyboard on table A each time they imagined arriving at a new location. Imagined action duration was therefore recorded for each distance. The experimenter followed the participants’ actions on the computer and removed the rolls to help the participants know how many rolls remained to be moved.

### Procedure

All participants were asked to move the toilet rolls with their hands or with a tool, in both real and imagery situations, as if they had come back from the supermarket and had to put away their purchases. Before each condition and situation, participants performed at least three practice trials. First, the experimenter carried out the first trial while explaining what he/she were doing. Second, the participant carried out the second trial with guidance from the experimenter. Finally, the participants performed the third trial and the experimenter only intervened if they made an error. If there was an error, the participant started again until he/she succeeded. After the training, the participants were asked to perform a trial for each quantity of rolls, each situation (real vs. imagined) and each condition (hands vs. tool), with 24 trials. The order of the trials between situations and conditions was balanced, leading to the following four orders: (1) hands—real, tool—real, hands—imagined, tool—imagined; (2) tool—real, hands—real, tool—imagined, hands—imagined; (3) hands—imagined, tool—imagined, hands—real, tool—real; (4) tool—imagined, hands—imagined, tool—real, hands—real. The sequence for each quantity of rolls was also counterbalanced by using two orders: 8, 24, 12, 20, 4, 16 vs. 16, 4, 20, 8, 12, 24. Therefore, there were eight possible orders (4 situations and help orders and 2 roll quantity orders), and 6 participants (3 individuals with schizophrenia and 3 non-clinical individuals) were distributed in each order. Finally, neuropsychological tests as well as semi-structured interviews (i.e., PANSS vs. MINI) were conducted.

### Main outcomes measures

We recorded the movement durations for each distance (AB, BA, AC, and CA) for all trials. We added these durations to obtain a total movement duration for each situation (real and imagined), for each condition (hands and tool), and for each quantity of rolls (4, 8, 12, 16, 20, and 24), which we named movement durations.

Following Osiurak et al.^3^, we computed a speed gain related to imagery in case of asymmetry between real vs. imagined durations. We first calculated the speed by dividing the distance (in meters) by the movement duration (in seconds). Then, we subtracted the real situation from the imagined situation. A positive score indicated that the participants took less time to imagine the actions (imagined movements) compared to the execution of the actions (real movements). This was computed for each condition. In addition, we had also decomposed the distances for tool use: the AC distance corresponding to the action of getting the tool (i.e., cost associated with getting the tool) and the AB distance corresponding to the action of the tool (i.e., benefit associated with tool-use action). Therefore, we investigated speed gain in four different conditions: Hands, Tool, Tool/AB distance, Tool/AB distance.

### Data analysis plan

All the analyses and the results reported in this study are original. Statistical analyses were performed with JASP software (version 0.14.0.0). Clinical, bodily, neuropsychological, movement durations, and speed gain data were tested for normal distribution with skewness and kurtosis indexes. No normal distribution was considered when absolute values for skewness and kurtosis were greater than 3 and 20, respectively^53^. Except the duration measurement during the revised shopping test, all measures for each experimental condition were below these values. Student’s *t* tests were performed for data with a normal distribution, and Mann–Whitney tests for data with a non-normal distribution. The first analysis compared sociodemographic, bodyweight, clinical, and neuropsychological data between individuals with schizophrenia and healthy control participants (Table 1). Then, the movement durations were analyzed with a four-way ANOVA with Group (healthy control vs. schizophrenia) as between subjects factor, Situation (imagined vs. real), Condition (hands vs. tool), and Rolls (4 vs. 8 vs. 12 vs. 16 vs. 20 vs. 24) as within subjects factors. In case of asymmetry between real vs. imagined durations, the imagery-related speed gain was computed and was tested by a three-way ANOVA with Group (healthy control vs. schizophrenia) as between subjects factor, Condition (hands vs. tool vs. tool/distances AB vs. tool/distances AC), Rolls (4 vs. 8 vs. 12 vs. 16 vs. 20 vs. 24) as within subjects factors. For some analyses of variance, the Mauchly test of sphericity was significant; a Greenhouse–Geiser correction was used. Effect sizes are presented using the partial squared eta measure *η*²_*p*_^54^ and interpreted according to Cohen’s D^55^. The level of significance was set to *p* < .05 and corrected using the Bonferroni procedure for post hoc multiple comparisons where necessary. Pearson correlations were conducted between imagery-related speed gain and negative symptoms (i.e., according to the subdimension of negative symptoms of PANSS and to the PANSS Amotivation factor). A Benjamini–Yekutieli correction was performed to control for false discovery rate of multiple tests^56^.

### Reporting summary

Further information on research design is available in the Nature Research Reporting Summary linked to this article.

## Supplementary information

Reporting Summary

## Data Availability

The data from this study are available from the corresponding author upon reasonable request.
