# Supplementary material for: Getting a tool gives wings even in schizophrenia: underestimation of tool-related effort in a motor imagery task
Source: NPJ Schizophr. 2021 Sep 15;7:45. doi: 10.1038/s41537-021-00175-y (PMC8443579; doi:10.1038/s41537-021-00175-y)
Supplement: Supplementary file 1 — Reporting Summary [file 41537_2021_175_MOESM1_ESM.pdf]

nature portfolio

Corresponding author(s): NPJSCHZ-00665R DBPR

Last updated by author(s): Jul 20, 2021

Reporting Summary

Nature Portfolio wishes to improve the reproducibility of the work that we publish. This form provides structure for consistency and transparency in reporting. For further information on Nature Portfolio policies, see our Editorial Policies and the Editorial Policy Checklist.

Statistics

For all statistical analyses, confirm that the following items are present in the figure legend, table legend, main text, or Methods section.

n/a	Confirmed	
☐	☒	The exact sample size (*n*) for each experimental group/condition, given as a discrete number and unit of measurement
☐	☒	A statement on whether measurements were taken from distinct samples or whether the same sample was measured repeatedly
☒	☐	The statistical test(s) used AND whether they are one- or two-sided<br>*Only common tests should be described solely by name; describe more complex techniques in the Methods section.*
☐	☒	A description of all covariates tested
☐	☒	A description of any assumptions or corrections, such as tests of normality and adjustment for multiple comparisons
☐	☒	A full description of the statistical parameters including central tendency (e.g. means) or other basic estimates (e.g. regression coefficient) AND variation (e.g. standard deviation) or associated estimates of uncertainty (e.g. confidence intervals)
☒	☐	For null hypothesis testing, the test statistic (e.g. *F*, *t*, *r*) with confidence intervals, effect sizes, degrees of freedom and *P* value noted<br>*Give P values as exact values whenever suitable.*
☒	☐	For Bayesian analysis, information on the choice of priors and Markov chain Monte Carlo settings
☒	☐	For hierarchical and complex designs, identification of the appropriate level for tests and full reporting of outcomes
☐	☒	Estimates of effect sizes (e.g. Cohen's *d*, Pearson's *r*), indicating how they were calculated

Our web collection on statistics for biologists contains articles on many of the points above.

Software and code

Policy information about availability of computer code

Data collection	we used OpenSesame 3.0.7 software for data collection
Data analysis	we used JASP software (version 0.14.0.0) for data analysis

For manuscripts utilizing custom algorithms or software that are central to the research but not yet described in published literature, software must be made available to editors and reviewers. We strongly encourage code deposition in a community repository (e.g. GitHub). See the Nature Portfolio guidelines for submitting code & software for further information.

Data

Policy information about availability of data

All manuscripts must include a data availability statement. This statement should provide the following information, where applicable:

- Accession codes, unique identifiers, or web links for publicly available datasets
- A description of any restrictions on data availability
- For clinical datasets or third party data, please ensure that the statement adheres to our policy

The data from this study are available from the corresponding author upon reasonable request.

Field-specific reporting

Please select the one below that is the best fit for your research. If you are not sure, read the appropriate sections before making your selection.

☐ Life sciences ☒ Behavioural & social sciences ☐ Ecological, evolutionary & environmental sciences

For a reference copy of the document with all sections, see nature.com/documents/nr-reporting-summary-flat.pdf

Behavioural & social sciences study design

All studies must disclose on these points even when the disclosure is negative.

Study description	quantitative methods
Research sample	The aim of the study was to compare individuals with schizophrenia to non-clinical individuals on the question of effort. For these reasons we decided to recruit a group of individuals with a diagnosis of schizophrenia (N = 25) and a group of non-clinical control individuals (N = 25), matched for age, education and gender.
Sampling strategy	With α=.05, power of 80%, for a 2x2x2x6 ANOVA, the sample size was 10 participants per group to detect a moderate effect size, using G*Power.
Data collection	Duration data were collected using OpenSesame software and bluetooth keyboards.<br>Clinical, body, and neuropsychological data, we recorded data with pen and paper.
Timing	August 2018 to March 2019
Data exclusions	two participants (one patient and one healthy control) were excluded because they did not complete the experiment, their data have been excluded.
Non-participation	two participants (one patient and one healthy control) were excluded because they did not to complete the experiment.
Randomization	participants were not allocated into experimental groups. the participants have achieved all the conditions (factors within subjects). Only the order of the conditions was a random factor; we included it as a covariate in the preliminary statistical analyses.

Reporting for specific materials, systems and methods

We require information from authors about some types of materials, experimental systems and methods used in many studies. Here, indicate whether each material, system or method listed is relevant to your study. If you are not sure if a list item applies to your research, read the appropriate section before selecting a response.

Materials & experimental systems

n/a	Involved in the study
☒	☐ Antibodies
☒	☐ Eukaryotic cell lines
☒	☐ Palaeontology and archaeology
☒	☐ Animals and other organisms
☐	☒ Human research participants
☒	☐ Clinical data
☒	☐ Dual use research of concern

Methods

n/a	Involved in the study
☒	☐ ChIP-seq
☒	☐ Flow cytometry
☒	☐ MRI-based neuroimaging

Human research participants

Policy information about studies involving human research participants

Population characteristics	see above
Recruitment	Patients with a diagnosis of schizophrenia were recruited from the hospital's inpatient units and day centers.<br>Non-clinical individuals were recruited from a list of volunteers for studies proposed by the hospital.
Ethics oversight	CPP Sud Méditérannée III

Note that full information on the approval of the study protocol must also be provided in the manuscript.
